# The effect of a micronutrient powder home fortification program on anemia and cognitive outcomes among young children in rural China: a cluster randomized trial

**DOI:** 10.1186/s12889-017-4755-0

**Published:** 2017-09-25

**Authors:** Renfu Luo, Ai Yue, Huan Zhou, Yaojiang Shi, Linxiu Zhang, Reynaldo Martorell, Alexis Medina, Scott Rozelle, Sean Sylvia

**Affiliations:** 1 China Center for Agricultural Policy, School of Advanced Agricultural Sciences, Peking University, Beijing, China; 2 Center for Experimental Economics in Education, Shaanxi Normal University, 620 Chang’an Road West, Xi’an, 710119 China; 3 West China School of Public Health, Sichuan University, Chengdu, China; 4 Center for Chinese Agricultural Policy, Chinese Academy of Sciences, Beijing, China; 5 Rollins School of Public Health, Emory University, Atlanta, USA; 6 Freeman Spogli Institute for International Studies, Stanford University, Stanford, USA; 7 Gillings School of Global Public Health, University of North Carolina at Chapel Hill, Chapel Hill, USA

**Keywords:** Micronutrient supplementation, Anemia, Cognition, Early childhood

## Abstract

**Background:**

Anemia early in life has been associated with delayed cognitive and motor development. The WHO recommends home fortification using multiple micronutrient powders (MNPs) containing iron as a strategy to address anemia in children under two. We evaluated the effects of a program freely distributing MNP sachets to caregivers of infants in rural China.

**Methods:**

We conducted a cluster-randomized controlled trial in Shaanxi province, enrolling all children aged 6–11 months in target villages. Following a baseline survey, investigators randomly assigned each village/cluster to a control or treatment group. In the treatment group, caregivers were instructed to give MNPs daily. Follow-up was after 6, 12, and 18 months of intervention. Primary outcomes were hemoglobin concentrations and scores on the Bayley Scales of Infant Development.

**Results:**

One thousand, eight hundred and-two eligible children and their caregivers were enrolled. At baseline 48% (870) of children were anemic and 29% (529) were developmentally delayed. Six hundred and-ten children (117 villages) were assigned to the control group and 1192 children (234 villages) were assigned to the treatment group. Assignment to the treatment group was associated with an improvement in hemoglobin levels (marginal effect 1.77 g/L, 95% CI 0.017–3.520, *p*-value = 0.048) and cognitive development (marginal effect 2.23 points, 95% CI 0.061–4.399, p-value = 0.044) after 6 months but not thereafter. There were no significant effects on motor development. Zero effects after the first 6 months were not due to low compliance, low statistical power, or changes in feeding behavior. Hemoglobin concentrations improved in both the treatment and control groups over the course of the study; however, 22% (325) of children remained anemic at endline, and 48% (721) were cognitively delayed.

**Conclusions:**

Providing caregivers with MNP sachets modestly hastened improvement in hemoglobin levels that was occurring absent intervention; however, this improvement did not translate into improved developmental outcomes at endline.

**Trial registration:**

ISRCTN44149146; prospectively registered on 15th April 2013.

## Background

Despite a spectacular rise in well-being in China over the past three decades, a large number of individuals remain in poverty. As a result of this poverty and associated factors, under-nutrition remains stubbornly high, particularly among children in China’s poor rural areas. Although rates of stunting and wasting are low, a large fraction of children in poor regions suffer from micronutrient deficiencies [1–3]. By far, the most common of these is iron deficiency [4–6]. According to data from China’s Food and Nutrition Surveillance System, anemia rates among young children under 2 years of age in 2005 were 30–40% in poor rural areas nationally [3, 7–9]. More geographically-focused studies have shown anemia rates higher than 50% in some areas [9–12]. Given that there are more than 8 million children born each year in rural areas, this implies that roughly 3 million children under 2 years old in rural China are anemic [13]. In China, it is postulated that 90% of anemia in infants and young children results from iron deficiency [14].

Anemia in early childhood is known to be particularly pernicious as it can serve to perpetuate poverty by keeping children from reaching their developmental potential. In the short run, anemia and iron deficiency in the first years of life is a major risk factor for cognitive, physical and emotional delays [15–23]. In the longer run, it can negatively affect educational attainment and even reduce earnings in adulthood [24–27]. These consequences may be irreversible, even if anemia is corrected later in childhood [24–28].

In settings with high prevalence of anemia (>20%) among children under 2 years of age, the WHO recommends home fortification of foods with multiple micronutrient powders (MNPs) [29]. This recommendation is based on a Cochrane review of eight randomized controlled trials that concluded MNPs to be effective in improving iron status and reducing anemia [30]. A more recent meta-analysis including several more recent studies also concluded that MNPs were effective in increasing hemoglobin concentration, but also noted substantial heterogeneity in effects across geographical settings [31]. In a recent cluster-randomized trial in Colombia, investigators found that MNPs were ineffective at reducing anemia or improving cognitive outcomes [31, 32]. They attribute the lack of efficacy of MNPs to the particular evolution of anemia with age among the Colombian population (early peak at under 1 year and rapid decline thereafter) and to a declining relative contribution of iron deficiency to remaining anemia as children age in Colombia. These characteristics of anemia set Colombia apart from settings where MNPs have been effective. In addition to geography, existing trials have also varied in aspects of study design including the age of targeted children, the duration of supplementation, and the timing of endline measurements [31].

Following the recommendations of the WHO, China’s government has announced intentions to implement a program to distribute MNPs through local clinics in nationally-designated poverty counties [33]. The program has already been initiated in select areas; however, the effectiveness of MNPs has yet to be rigorously evaluated in China.

We present the results of a large-scale effectiveness trial of home fortification using MNPs in villages in rural western China. In line with the national program, we designed the trial to begin providing supplements when children were 6–11 months old and track outcomes every 6 months over 18 months. In addition to hemoglobin and anemia, we evaluate effects on cognitive and psychomotor development measured using the Bayley Scales of Infant Development (BSID), given the well-established link between early childhood anemia and cognitive and physical delays [15–23]. Such developmental delays in early childhood can have significant consequences long term. Studies have linked early childhood interventions to increased college attendance, employment, earnings as well as reductions in teen pregnancy and criminal activity [34–36].

## Methods

### Study design, sampling frame, and participants

We carried out a cluster-randomized controlled trial in southern Shaanxi Province. Shaanxi is a relatively poor province in Western China and ranks 19 out of 31 provinces nationally in terms of wealth. To identify the sample, we first selected 174 townships from 11 nationally-designated poverty counties in southern Shaanxi. All townships in each county were included except (1) the one township per county that housed the county seat and (2) those townships that did not have any villages with at least 800 people. These exclusion criteria were chosen to ensure a rural sample and increase the likelihood that sampled villages had a sufficient number of children 6–11 months of age.

Within each township, sample villages were selected in April, 2013. We used official government data to compile a list of villages in each township. We then randomly selected 2 villages from the list in each township. An additional village was selected in randomly chosen townships to meet power requirements. Our final sample consisted of 351 villages. A list of all registered births over the past 12 months was obtained from the local family planning official in each village. All children in our desired age range (6–11 months) were enrolled in the study. A second cohort of children in our desired age range (6–11 months) was enrolled from the same sample villages in October 2013. Overall, the baseline sample included 1802 children.

Following the baseline survey for the first cohort, villages were allocated to an MNP arm or a control arm. For the first 6 months of the study, a random half of the MNP arm also received text message reminders. (Results are qualitatively similar excluding this group – this analysis is reported in Table 6 in Appendix.) The intervention period lasted for 18 months for both cohorts (from April 2013 to October 2014 for cohort 1 and from October 2013 to April 2015 for cohort 2).

### Randomization and masking

Following the baseline survey, we randomly assigned 117 villages to the control group and 234 villages to the micronutrient group using computer code (in Stata version 12). Randomization was stratified within counties.

We did not use a placebo in the control group for ethical and practical reasons. Caregivers were not aware that they were in an RCT.

### Interventions

We used a Heinz-produced MNP called “NurtureMate.” The powder is tasteless and each daily-dose sachet contains 6 mg of iron as well as zinc, vitamins A, C, D, B_1,_ B_2_,B_6_, B_12_, and folic acid (Table 1). NurtureMate is recommended for infants and toddlers aged 6–36 months. The full composition of the NurtureMate sachets was based on the general standard for complementary food supplements in China [37] and was approved for use in the national program to distribute MNPs through local clinics in poverty counties (piloting for the national program began after the start of this study, but not in our study regions).

**Table 1:** Composition of NurtureMate home fortification powders^a^

Nutrients	Unit	Average content per packet (1 g)	RNI or AI in China (6–11 months) per day^b^	RNI or AI in China (12–36 months) per day	RNI or AI (%)	
					6–11 month	12–36 month
Iron (ferrous lactate)	mg	6.0	10	12	60%	50%
Zinc (zinc sulfate)	mg	4.80	8	9	60%	53%
Vitamin A	μgRE	200	400	500	50%	40%
Vitamin C	mg	50.0	50	60	100%	83%
Vitamin D	μg	5.0	10	10	50%	50%
Vitamin E	mg	1.55	3	4	52%	39%
Vitamin B1	mg	0.30	0.3	0.6	100%	50%
Vitamin B2	mg	0.50	0.5	0.6	100%	83%
Vitamin B6	mg	0.30	0.3	0.5	100%	60%
Vitamin B12	μg	0.5	0.5	0.9	100%	56%
Folic acid	μg	66	80	150	83%	44%
Niacin	mg	3.0	3	6	100%	50%
Energy	kJ	15			–	–
Protein	g	0			–	–
Fat	g	0			–	–
Carbohydrate	g	0.9			–	–

^a^NurtureMate can be mixed in with either water or complementary foods, such as porridge, and is recommended for children aged 6–36 months. Recommended consumption is 5–7 sachets per week (one per day)

^b^RNI: Recommended Nutrient Intake. AI: Adequate Intakes

Caregivers in the MNP arm were given a 6-month supply of sachets every 6 months. At the first distribution they were also given information about the causes and consequences of anemia along with oral and written instructions on how to use the powder, specifically to give one packet per day mixed with the child’s food. During the first distribution, households were also given a plastic storage envelope in which to store the empty NurtureMate sachets. Thus, the treatment was designed to include components of a policy distributing MNP sachets (including information on sachets and use and interaction every 6 months through sachet distribution). This allows us to identify the effects of such a policy, beyond the impact of MNPs themselves.

### Data collection

The research team conducted four rounds of data collection for each cohort. The initial baseline survey was conducted in April 2013 for cohort 1 and in October 2013 for cohort 2. During each survey round, nurses from Xi’an Jiaotong Medical School collected data on hemoglobin concentrations from all children. Hemoglobin concentrations were measured using HemoCue Hb 201+ finger prick systems (HemoCue Ltd., Ängelholm, Sweden).

Teams of enumerators collected socioeconomic data from study households. Each child’s primary caregiver was identified and administered a survey on child, parent and household characteristics including each child’s gender, birth order, maternal age and education. Each child’s age was obtained from his or her birth certificate. The primary caregiver was identified by each family as the individual most responsible for the child’s care (typically the child’s mother or grandmother).

As part of the household survey in each round of data collection, the primary caregiver was asked about the diets of children in the household. Caregivers were asked whether the child’s diet during the previous day included items from each of seven food categories: meat or fish; beans, nuts or seeds; milk products; eggs; fruits and vegetables rich in Vitamin A; other fruits and vegetables; and grains. We designed questions about food consumption as a series of yes/no questions to minimize measurement error. Detailed questions were also asked about breastfeeding and formula consumption.

Enumerators tallied unused and empty packets to assess compliance in follow-up surveys. Although there is some potential for measurement error, this compliance measure is unlikely to be biased because households and enumerators had little incentive to misreport use of MNP sachets. Further, measures based on counting empty sachets were consistent with the count of unopened sachets and with responses given by caregivers about sachet use.

All children were also administered the Bayley Scales of Infant Development (BSID) Version I, an internationally-scaled test of children’s cognitive and motor development [38]. The BSID is widely used in research on early childhood development, and is often considered a “gold standard” for assessing early childhood development [39]. The test was formally adapted to the Chinese language and environment in 1992 and scaled according to an urban Chinese sample [40, 41]. Following other published studies that use the BSID to assess child development in China [42–44], it was this officially adapted version of the test that was used in this study. All BSID enumerators attended a week-long training course on how to administer the BSID, including a 2.5 day experiential learning program in the field. The test was administered in the household using a standardized set of toys and detailed scoring sheet. The BSID takes into consideration each child’s age in days, as well as whether he or she was premature at birth. These two factors, combined with the child’s performance on a series of tasks using the standardized toy kit, contribute to the establishment of the two sub-indices: MDI, which evaluates memory, habitation, problem solving, early number concepts, generalization, classification, vocalization and language to produce a measure of cognitive development; and PDI, which evaluates gross motor skills (rolling, crawling and creeping, sitting and standing, walking, running and jumping) and fine motor skills to produce a measure of psychomotor development [45]. For the analysis, raw scores for each of these sub-indices are normalized using the distribution of scores in a reference group of healthy children in China of the same age supplied by the testing company. These normalized scores have an expected mean of 100 and a standard deviation of 16, with impairment for each index defined as a score below 80.

### Statistical analysis

We analytically calculated the sample size for the study to detect a 0.2 standardized effect on MDI scores. Based on previous studies [41, 46–49], we assumed an intracluster correlation of 0.1 and that baseline scores would account for 50% of the variation in scores at endline. We further assumed that there would be four sample children per village after attrition. Based on these parameters, we calculated that we required 112 villages/group to detect a standardized effect of 0.2 at 80% power given a significance level of 0.05. We added five villages to each group to overpower the study and to account for potential attrition as funding allowed.

The primary outcomes of the study were hemoglobin (measured in g/L) and the two subindices of the BSID: the MDI and PDI. We also report effects on anemia (using the WHO definition of altitude-adjusted Hb concentration below 110 g/L) [50] for comparability with existing lieterature, but note that there is uncertainty over the appropriateness of hemoglobin thresholds to define anemia status [51].

We estimated the treatment effects on our primary outcomes using intent-to-treat (ITT) analysis. We used multiple regression analysis to estimate ITT impacts. When estimating effects on hemoglobin concentration, we adjust for child gender and age and also control for Hb test machine fixed effects. When estimating effects on BSID scores, we control for child gender, age, caregiver engagement (playing/singing yesterday) and BSID tester (enumerator) fixed effects. All regressions control for cohort fixed effects and county (strata) fixed effects to account for the randomization procedure. In all analyses, we account for clustering within villages (the level of randomization) using Huber-White cluster-adjusted standard errors.

In order to accommodate partial compliance and to estimate the effect of MNP sachet use on hemoglobin concentrations and development scores, we use an instrumental variables approach to estimate the average treatment on the treated effect [52]. Specifically, we instrument the total number of sachets consumed (as of each survey round) with a variable indicating whether the child was assigned to the MNP group (the instrumental variable). This allows us to estimate the causal effect of MNP sachet consumption, assuming a linear relationship.

In order to aid in the interpretation of effects on the primary outcomes of the study (hemoglobin and BSID scores), we also assess impacts of the MNP intervention on feeding behavior. The MNP intervention may cause caregivers to feed children more nutritious foods if, for example, the intervention increased awareness of or attention to nutrition generally. On the other hand, it could also “crowd-out” more nutritious foods if MNPs are viewed by caregivers as a substitute for food sources of micronutrients. For each of the seven food categories, we estimate the ITT effects in the same manner as with the primary outcomes, but controlling only for cohort and county fixed effects.

Statistical analyses were conducted using STATA 13.0 (StataCorp, College Station, TX, USA). *P*-values below 0.05 are considered statistically significant.

## Results

**Fig. 1:**
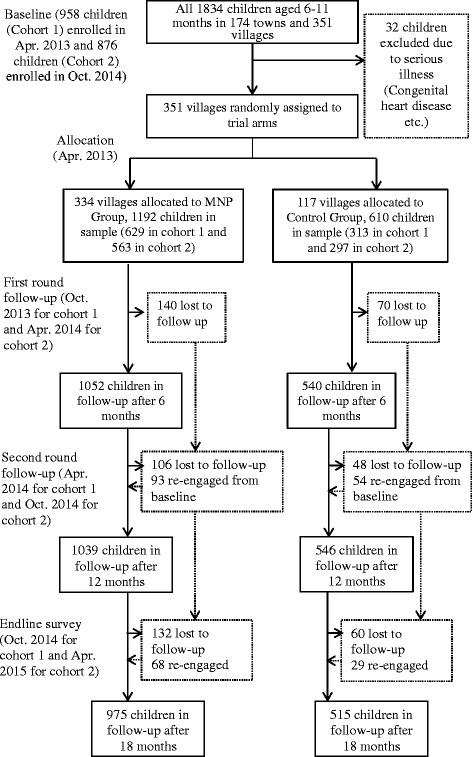
Trial profile. MNP = Micronutrient Powder

A total of 1834 children were enrolled at baseline across the two cohorts (Fig. 1). We excluded 32 children due to serious illnesses. Table 2 shows characteristics of children included in the analysis sample at baseline. Of the 1802 children, 1592 (88%) remained in the sample at 12–17 months of age, 1585 (88%) remained in the sample at 18–23 months of age, and 1490 (82%) remained in the sample at 24–29 months of age. No villages (clusters) were lost. Attrition rates did not vary significantly between the experimental arms (Table 7 in Appendix).

**Table 2:** Baseline characteristics by experimental arm (*N* = 1802)

Characteristics	Control Group (*n* = 610)	Free MNP Group^1^ (*n* = 1192)	*P* ^*2*^
Social economic status			
Age in months^3^	9.46 ± 1.90	9.50 ± 1.83	0.75
Girls (%)	49.0 (299)	46.5 (544)	0.26
Low birth weight (%)	4.4 (27)	4.7 (56)	0.80
First birth (%)	62.3 (380)	62.7 (747)	0.88
Families received social security support (%)	24.4 (149)	23.2 (276)	0.45
Caregiver and mother characteristics			
Mother is primary caregiver (%)	79.2 (483)	81.8 (975)	0.31
Maternal education ≥9 years (%)	78.4 (478)	82.3 (981)	0.07
Maternal age (year)	26.2 ± 4.3	26.5 ± 4.7	0.25
Child feeding practices			
Ever breastfed (%)	87.7 (535)	89.3 (1064)	0.36
Exclusive or predominant breastfeeding <6 Months (%)	37.7 (230)	37.8 (451)	0.97
Still breastfed ≥12 Months (%)	39.2 (239)	37.3 (444)	0.14
Ever formula-fed (%)	63.4 (387)	66.4 (792)	0.07
Supplementary feeding after six months (%)	65.9 (402)	65.3 (778)	0.87
Parenting practices			
Played with baby yesterday with toys (%)	49.8 (304)	46.7 (557)	0.12
Sang song to baby yesterday (%)	29.0 (177)	28.7 (342)	0.78
Child nutrition status			
Hemoglobin concentration (g/L) ^4^	109.3 ± 13.0	109.1 ± 12.5	0.71
Anemia prevalence (%)^4^	49.3 (297)	48.5 (573)	0.88
Child development status			
MDI test score^5^	97.2 ± 17.0	96.6 ± 16.9	0.87
PDI test score^6^	90.1 ± 18.0	90.1 ± 16.7	0.85

Data are presented as mean ± SD or % (*n*) for categorical variables

^1^MNP = Multiple Micronutrient Powder

^2^ P-values from tests of the null hypothesis of no difference between MNP and control groups, adjusted for cohort and county fixed effects and clustering within villages

^3^ Data missing for 4 children in the free MNP group

^4^Data missing for 7 children in control group and 10 in the free MNP group

^5^Data missing for 1 child in control group and 4 in the free MNP group

^6^Data missing for 2 children in control group and 5 in the free MNP group

^7^Data missing for 4 children in control group and 2 in the free MNP group

At baseline, the average hemoglobin concentration of 6–11 month olds was 109 g/L. Nearly half of the sample children (49%) were anemic. Fourteen percent of children were delayed in their cognitive development (MDI score < 80) and 24% of children were delayed in their psychomotor development (PDI score < 80); 29% of children had either cognitive or psychomotor delay.

The average number of sachets consumed each month in the MNP group over the course of the study was 14 (SD 11.6—Table 8 in Appendix). The average number of sachets consumed declined slightly across the survey waves from 16 sachets per month in the first 6-month period to 13 sachets in the third.

**Fig. 2:**
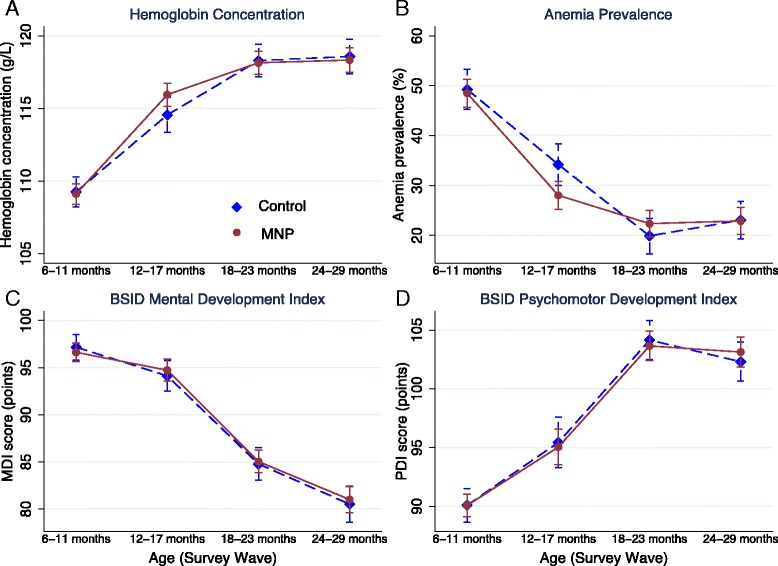
Hemoglobin concentration (g/L), anemia prevalence (%), mental and psychomotor Development of sample children by experimental arm and age/survey wave. MNP = Multiple Micronutrient Powders, MDI = Bayley Mental Development Index, PDI = Bayley Psychomotor Development Index

Figure 2 shows the average hemoglobin concentration (Panel A) and anemia prevalence (Panel B) with 95% confidence intervals over the four survey rounds, separately for the MNP and control group. Overall, there is a steady increase in hemoglobin levels (and a steady decrease in anemia rates) over the course of the study in both groups. In the control group, average hemoglobin rose from 109 g/L on average when children were 6–11 months old to 118 g/L when children were 24–29 months old. Corresponding anemia rates more than halved, but remained high (23%).

Trends in the MDI (Panel C) and PDI (Panel D) indices also are presented in Fig. 2. Over the course of the study there was a marked decline in average MDI scores from 97 to 81. The number of children with cognitive delay increased from 14% at 6–11 months of age to 49% at 24–29 months of age. In contrast to MDI, we find that average PDI scores increased over time, rising from 90 to 102.

Table 3 shows the estimated treatment effects of the MNP intervention on hemoglobin concentration, anemia status and the two BSID sub-indices based on the ITT analysis. For each outcome, we report impact estimates at 6, 12, and 18 months after the start of the intervention.

**Table 3:** Effects of multiple micronutrient powder provision on haemoglobin (Hb) concentration, anaemia prevalence, and Bayley mental and psychomotor development scores

	Hb concentration (g/L)^1^		Anemia prevalence (Hb <110 g/L)^1^		Bayley MDI (points)^2^		Bayley PDI (points)^2^	
	Marginal Effect (95% CI)	*P* value	Marginal Effect (95% CI)	*P* value	Marginal Effect (95% CI)	*P* value	Marginal Effect (95% CI)	*P* value
Effect of MNP distribution after 6 months (children 12–18 months of age)	1.769 (0.017–3.520)	0.048*	− 0.058 (− 0.127–0.010)	0.095	2.230 (0.061–4.399)	0.044*	− 0.266 (− 3.328–2.796)	0.864
Effect of MNP distribution after 12 months	− 0.115 (− 2.189–1.959)	0.913	0.034 (− 0.039–0.107)	0.357	0.487 (− 2.071–3.046)	0.708	− 0.461 (− 2.938–2.016)	0.715
Effect of MNP distribution after 18 months	0.127 (− 1.850–2.105)	0.899	− 0.001 (− 0.071–0.070)	0.982	0.828 (− 1.787–3.444)	0.534	0.084 (− 2.277–2.445)	0.944
Intracluster Correlation (ICC)^3^	0.09		–		0.08		0.02	
R-squared	0.14		0.10		0.27		0.21	
Observations	6092		6092		6204		6186	

^1^Intention to treat analysis, and regression estimates from multiple linear models adjusted for gender, age, Hb test machine and county fixed effects. Standard errors are clustered at the village level. Hb data missing for 349 observations (17 in baseline, 115, 144 and 73 in 6 months, 12 months and 18 months follow-up)

^2^Intention to treat analysis, and regression estimates from multiple linear models adjusted for gender, age, parenting practice (whether caregiver used toys played with child the previous day, sang to child previous day), BSID tester and county fixed effects. Standard errors are clustered at the village level. MDI data missing for 214 observations (5 in baseline, 101, 88 and 20 in 6 months, 12 months and 18 months follow-up), and PDI data missing for 233 children (7 in baseline, 102, 98 and 26 in 6 months, 12 months and 18 months follow-up)

^3^Intracluster correlation (ICC) at endline (final wave). No ICC is reported for anemia as this is a binary outcome

*p-value < 0.05 significant

The effect of the MNP intervention on average hemoglobin concentration was 1.77 g/L (*p* = 0.048; 95% CI 0.02—3.52 g/L) after 6 months (when children were 12 to 17 months of age). This effect did not persist, however. At 12 and 18 months after the start of the intervention, estimated effects are not statistically different from zero. After 6 months of the intervention, we find a reduction in anemia of six percentage points (18%); however, this does not reach statistical significance at 5%. We find no effect on anemia status after 12 or 18 months of intervention.

The effect of the MNP intervention on cognition (MDI) shows a pattern similar to that of hemoglobin concentration. After 6 months of supplementation, the effect of MNP consumption on the MDI sub-index was 2.23 points (*p* = 0.044; 95% CI 0.061—4.399), equal to 0.13 SD of the baseline distribution in the control group. We did not detect a significant effect on MDI after 12 or 18 months (*p* > 0.5). We did not detect any significant effect on PDI scores in any of the time periods. No significant harms were reported.

We find no significant effects for any time period when estimating effects on the probability of delay (MDI or PDI < 80) rather than the continuous MDI and PDI measures. These results are reported in Tables 9 and 10 in Appendix.

Table 4 reports the effects of MNP consumption estimated using instrumental variables (IV) in order to account for imperfect compliance. We estimate that each additional sachet consumed during the first 6 months of the program increased hemoglobin concentration by 0.023 g/L (p = 0.04; 95% CI 0.001—0.045). This estimate suggests that with full compliance (the recommended one sachet per day), MNPs increased hemoglobin concentration by 4.14 g/L (180 days × 0.023 g/L) assuming a linear relationship. The implied increase is 2.07 g/L with 50% compliance. Similar to the ITT estimates, however, we do not find a significant effect of MNPs on hemoglobin concentrations after 12 or 18 months even after adjusting for compliance. The analysis does not find any significant effects on anemia prevalence in any of the time periods after the baseline.

**Table 4:** Dose–response effects of multiple micronutrient powder sachet consumption on heamoglobin (Hb) concentration, anaemia prevalence, and Bayley mental and psychomotor development scores

	Hb concentration (g/L)^1^		Anemia prevalence (Hb <110 g/L)^1^		Bayley MDI (points)^2^		Bayley PDI (points)^2^	
	Marginal Effect (95% CI)	P value	Marginal Effect (95% CI)	P value	Marginal Effect (95% CI)	P value	Marginal Effect (95% CI)	P value
Effect of MNP sachet consumption after 6 months (dose–response per sachet)	0.023 (0.001–0.045)	0.040*	− 0.001 (− 0.002–0.0001)	0.087	0.028 (0.002–0.054)	0.034*	− 0.001 (− 0.038–0.035)	0.943
Effect of MNP sachet consumption after 12 months	− 0.002 (− 0.026–0.022)	0.883	0.000 (− 0.001–0.001)	0.427	0.007 (− 0.022–0.035)	0.640	− 0.004 (− 0.031–0.023)	0.774
Effect of MNP sachet consumption after 18 months	0.002 (− 0.022–0.027)	0.861	− 0.000 (− 0.001–0.001)	0.977	0.015 (− 0.016–0.047)	0.346	0.003 (− 0.026–0.031)	0.843
R-squared	0.14		0.10		0.26		0.21	
Observations	6003		6003		6113		6096	

^1^ Average Treatment on the Treated analysis, using the treatment assignment as an instrumental variable for the total number of sachets used in the preceding six months. Regression estimates from multiple linear models adjusted for gender, age, Hb test machine and county fixed effects. Standard errors are clustered at the village level. Hb data missing for 349 children (17 in baseline, 115, 144 and 73 in 6 months, 12 months and 18 months follow-up) and feeding behavior data missing for 206 children (74, 91 and 41 in 6 months, 12 months and 18 months follow-up)

^2^ Average Treatment on the Treated analysis, using the treatment assignment as an instrumental variable for the total number of sachets used in the preceding six months. Regression estimates from multiple linear models adjusted for gender, age, parenting practice (whether caregiver used toys played with child the previous day, sang to child previous day), BSID tester and county fixed effects. Standard errors are clustered at the village level. MDI data missing for 214 children (5 in baseline, 101, 88 and 20 in 6 months, 12 months and 18 months follow-up), PDI data missing for 233 children (7 in baseline, 102, 98 and 26 in 6 months, 12 months and 18 months follow-up), and feeding behavior data missing for 206 children (74, 91 and 41 in 6 months, 12 months and 18 months follow-up)

*p-value < 0.05 significant

For MDI, the IV analysis suggests that each additional sachet consumed increased MDI by 0.028 points (*p* = 0.034; 95% CI 0.002—0.054) at 6 months. By 12 months after the start of the intervention, however, this effect also subsided. The analysis did not detect any effects on MDI at 12 or 18 months. We did not detect any significant effect on PDI scores in any of the time periods.

Results for feeding behavior are presented in Table 5. At baseline (when children were 6–11 months of age), we find that few children in either group were fed iron rich foods such as meat (around 18% were fed meat or fish) or legumes (around 18% report feeding beans, nuts and seeds). Most children were fed grains (approximately 86%), milk products (around 58%), and fruits and vegetables rich in Vitamin A (around 55%) the previous day. Eggs were fed to around 34% of children and other fruits and vegetables were fed to 48% of children.

**Table 5:** Feeding of sample child by age and experimental arm^1^

Food fed the previous day:	6–11 months			12–17 months			18–23 months			24–29 months		
	Control Group (*n* = 285)	MNP Group^2^ (*n* = 549)	P^3^	Control Group (*n* = 514)	MNP Group^2^ (*n* = 989)	P^3^	Control Group (*n* = 510)	MNP Group^2^ (*n* = 988)	P^3^	Control Group (*n* = 511)	MNP Group^2^ (*n* = 968)	P^3^
Meat or fish	18.3 (52)	18.4 (101)	0.94	34.8 (179)	39.1 (386)	0.09	51.0 (210)	51.1 (505)	0.89	56.2 (287)	57.2 (553)	0.62
Beans, nuts, or seed products	17.9 (51)	18.0 (99)	0.90	26.9 (138)	26.6 (263)	0.78	36.5 (186)	35.1 (347)	0.45	42.3 (216)	38.6 (374)	0.13
Milk products	57.9 (165)	60.1 (330)	0.46	78.4 (402)	81.0 (801)	0.19	82.1 (417)	81.7 (806)	0.79	67.1 (343)	72.9 (704)	0.03*
Eggs	33.7 (96)	30.6 (168)	0.39	45.3 (233)	47.1 (465)	0.43	44.1 (225)	47.7 (471)	0.20	45.1 (230)	46.7 (452)	0.47
Vegetables and fruits rich in Vitamin A	55.3 (157)	54.8 (300)	0.84	73.0 (375)	78.9 (779)	0.02*	80.6 (411)	81.8 (807)	0.60	80.8 (413)	84.6 (819)	0.07
Other vegetables and fruits	47.7 (136)	45.0 (247)	0.44	59.0 (303)	61.3 (606)	0.43	69.8 (306)	68.5 (677)	0.71	69.2 (353)	67.1 (649)	0.32
Grains	85.6 (244)	86.3 (474)	0.77	94.8 (487)	95.2 (941)	0.78	97.8 (498)	97.4 (962)	0.55	97.9 (500)	99.1 (960)	0.07

Data are presented as % (*n*) for categorical variables

^1^ We only collected detail information of diet for cohort 2 in the baseline (child aged 6–11 months), but for child aged 12–30 months, we collected detailed information of diet for cohort 1 and cohort 2

^2^MNP = Multiple Micronutrient Powder

^3^ P-values from tests of the null hypothesis of no difference between MNP and control groups, adjusted for cohort and county fixed effects and clustering within villages *p-value < 0.05 significant

How feeding practices transition as children age is also evident in Table 5. Focusing on the responses of caregivers in the control group across the four survey waves, three patterns are of particular note. First, a dramatic overall change in diet occurs between when children are 6–11 months to when they are 12–17 months of age. There is a significant increase in the consumption of all food categories. This is not unexpected as this is a time when solid foods are introduced.

Second, there is an initial increase followed by a leveling off of consumption of grains, eggs, and fruits and vegetables. Nearly all children are consuming grains by 12–17 months. Around 45% of children are consuming eggs by 12–17 months. The consumption of fruits and vegetables is increasing until children are 18–23 months. By this time around 80% of children were fed vegetables or fruits rich in Vitamin A the previous day and around 70% had been fed other fruits and vegetables.

Finally, there is a gradual increase across the four waves in the consumption of iron-rich foods (meat and fish; beans, nuts and seeds). However, by 24–29 months consumption of beans, nuts and seeds remains below 50% and the consumption of meat and fish remains below 60%.

These patterns are largely consistent across both the control and intervention groups; in other words, we do not find that the intervention had important effects on feeding behavior. Although children in the intervention group consume significantly higher quantities both of vegetables and fruits rich in Vitamin A at 12–17 months, and of milk products at 24–29 months, there are few significant differences overall. There is no evidence that the intervention crowded out nutritious foods.

## Discussion and conclusion

In a large-scale cluster randomized trial of multiple micronutrient powders (MNPs) in rural China, we found that home fortification using MNPs significantly increased hemoglobin levels after 6 months of supplementation (when children were 12 to 17 months of age) but that hemoglobin levels were not significantly higher than the control group after 12 months of supplementation (when children were 18 to 23 months of age) or after 18 months of supplementation (when children were 24 to 29 months of age). During this period from 6 to 30 months of age, a time in which children’s diets are in transition, hemoglobin concentrations in the control group increased rapidly (although anemia levels remained high). The MNP intervention hastened this improvement during the first 6 months, however, these positive effects subsided thereafter as hemoglobin levels accelerated absent intervention as children aged from 12 to 17 months to 18–23 months of age.

The effects of the intervention on cognition (as measured by MDI scores on the BSID) followed a similar pattern of effects over time to that observed on hemoglobin concentration. We found a significant improvement on the MDI score at 6 months. However–although it would be reasonable to hypothesize a sustained or delayed effect of supplementation on cognitive development even after MNPs no longer affected hemoglobin–cognitive scores were also not significantly higher than the control group after 12 or 18 months of supplementation. MNPs had no detectable effect on motor development at any point during the trial.

Using an IV analysis to adjust for partial compliance, we find a similar pattern of results, suggesting that the lack of effects in later rounds was not due to reduced consumption of the MNP sachets. Moreover, nil effects after the 6 month mark were unlikely due to low power. The study retained power to detect small effects throughout the trial as evidenced by relatively tight confidence intervals on impact estimates in later rounds.

Although it is difficult to say what led to this lack of effects, we note that our results are strikingly similar to those of a recent trial testing MNPs in Colombia [31, 32]. The authors of the Colombia study suggest that the zero/low effects in that study may have been due to the changing nature of the causes of low hemoglobin and rapidly decreasing anemia rates over time as children (in the control group) age. As with our study, they find that hemoglobin concentrations in the control group increased substantially as children aged from 12 to 30 months. This is in contrast to less rapid increases in hemoglobin concentration (absent intervention) in other regions where trials of MNPs have been successful [53, 54]. We find no evidence that the MNP intervention changed food consumption suggesting that the lack of observed effects is not due to a crowding-out of nutritious foods.

Another possibility is that the amount of iron in the sachets used in this study (6 mg) was simply too low. Most previous trials of MNPs used sachets that contained 12.5 mg of iron [30, 31]. The amount of iron in the sachets was set at 6 mg in order to comply with regulations in China [37]. Sachets with similarly low iron content have been suggested for use in a national program distributing sachets through local health centers.

One major difference between this study and the proposed national program is that the national program would provide MNP sachets through local health centers while we delivered sachets to the homes of caregivers. The average effect of the national program is therefore likely to be smaller than what we find in this study with home delivery, assuming that take-up rates would be lower. The provision of sachets through local health centers may also lead to selective take-up, potentially to the disadvantage of anemic children most in need of intervention.

This study makes important contributions to the evidence base on the use of MNPs to address micronutrient deficiency among children. The main strength of the study is that it is an effectiveness trial designed to mimic the effects of a government program. Most existing studies of home fortification programs have employed weekly or biweekly visits from researchers to a tightly controlled researcher-implemented environment [55–58], an approach which would be costly to implement at scale. In our study, we delivered the NurtureMate every 6 months to the households, and left households alone to resume their regular routines.

This study also employed a more extensive data collection effort than previous trials. First, we evaluate effects on both anemia and measures of cognitive development. The three rounds of follow-up surveys at 6 month intervals also allow us to investigate how impacts of MNPs evolve over time. We are aware of only two other studies of micronutrient powders that collected as many rounds of follow-up information [53, 54].

Despite these strengths, our study does have several limitations. First, the observed attrition rate was relatively high: 28% of sample children were missing from at least one of the three survey rounds. Overall, however, 97% of the sample was followed-up at least once, which is high compared to previous studies. Attrition rates were similar between the treatment and control groups (as shown in Table 7 in Appendix), but results could have been affected if mechanisms driving attrition differed across groups. We find no evidence of this and do not believe that our findings are invalidated by attrition. A second limitation is that we were unable to conduct information on biomarkers beyond hemoglobin. Additional indicators of iron status, markers of inflammation and additional mineral and vitamin biomarkers would have been desirable. Third, the trial was conducted in one poor region of western China; results may not generalize to other settings. Finally, while we speculate above, we are unable to say definitively why MNPs were ineffective at reducing anemia rates as children aged past 1 year. A priority for future studies should be to better understand the drivers of anemia in young children and how these change as children age.

In sum, our results show that MNPs (using the formulation of China’s proposed national program) did not improve anemia or cognitive outcomes of children after 18 months of age. Although anemia rates appear to fall quickly as children age, they remained high at 2 years of age. Such results suggest that the government might consider raising the level of iron that is allowed in MNP supplements. More alarming is the high level of cognitive delay among 2 year olds. Given that the national program to distribute MNPs is unlikely to address high levels of cognitive delay among children, there is an urgent need for effective strategies to promote the development of young children in China’s rural areas. Without effective interventions, a large fraction of China’s children will be prevented from reaching their full potential.

## Appendix

**Table 6:** Effects of multiple micronutrient powder provision on heamoglobin (Hb) concentration, anaemia prevalence, and Bayley mental and psychomotor development scores by experimental assignment in first period

	Hb concentration (g/L)^1^		Anemia prevalence (Hb <110 g/L)^1^		Bayley MDI (points)^2^		Bayley PDI (points)^2^	
Trial arms compared	Difference (95% CI)	*P* value	Marginal effect (95% CI)	*P*- value	Difference (95% CI)	*P*- value	Marginal effect (95% CI)	*P*- value
Free vs control after 6 months follow-up^3^	1.840 (− 0.189–3.868)	0.075	− 0.048 (− 0.129–0.034)	0.252	3.025 (0.443–5. 606)	0.022*	− 1.624 (− 5.166–1.918)	0.368
Free vs control after 12 months^3^	− 0.502 (− 2.760–1.757)	0.662	0.026 (− 0.056–0.108)	0.535	1.978 (− 0.899–4.855)	0.177	− 0.846 (− 3.807–2.114)	0.574
Free vs control after 18 months^3^	− 0.718 (− 2.930–1.494)	0.524	0.026 (− 0.054–0.107)	0.523	2.808 (− 0.159–5.774)	0.064	− 0.162 (− 2.785–2.419)	0.902
Text vs control after 6 months^4^	1.715 (− 0.347–3.777)	0.103	− 0.069 (− 0.146–0.007)	0.075	1.423 (− 1.184–4.030)	0.284	1.061 (− 2.379–4.501)	0.544
Text vs control after 12 months ^4^	0.266 (− 2.220–2.752)	0.833	0.042 (− 0.046–0.129)	0.348	− 0.70 (− 3.788–2.389)	0.656	− 0.062 (− 2.886–2.763)	0.966
Text vs control after 18 months ^4^	0.936 (−1.419–3.290)	0.435	− 0.027 (− 0.110–0.056)	0.526	− 0.776 (− 3.90–2.348)	0.626	0.347 (− 2.600–3.294)	0.817
R-squared	0.14		0.10		0.27		0.21	
Observations	6092		6092		6204		6186	

^1^ Intention to treat analysis, and regression estimates from multiple linear models adjusted for gender, age, Hb test machine and county fixed effects. Standard errors are clustered at the village level

^2^ Intention to treat analysis, and regression estimates from multiple linear models adjusted for gender, age, parenting practice (whether caregiver used toys played with child the previous day, sang to child previous day), BSID tester and county fixed effects. Standard errors are clustered at the village level

^3^ “Free” group received free MNP sachets but not daily text message reminders

^4^ “Text” group received free MNP sachets and daily text message reminders for the first six months of the trial

**p*-value < 0.05 significant

**Table 7:** Comparison of attrition across MNP and control groups (N = 1802)

Dependent Variable:	Lost to follow up at least once (yes = 1, no = 0) ^1^	
Trial arms compared	Difference (95% CI)	*P* value
MNP vs control	0.018 (− 0.026–0.063)	0.412
R-squared	0.03	
Observations	1802	

^1^Regression estimates from multiple linear models adjusted for county fixed effects. Standard errors are clustered at the village level

**Table 8:** The average number sachets consumed monthly in MNP group

Intervention time	Free MNP Group^a^
	Average number of sachets consumed monthly
First 6 months intervention	15.73 ± 12.1
Between month 7 and 12 of intervention	14.1 ± 11.0
Between month 13 and 18 of intervention	12.9 ± 11.6
Average	14.3 ± 11.6

Data are presented as mean ± SD

^a^MNP = Multiple micronutrient powders

**Table 9:** Effects of multiple micronutrient powder provision on Bayley mental and psychomotor development delay

	Mental delay (MDI < 80)^1^		Psychomotor delay (PDI < 80)^1^	
	Marginal Effect (95% CI)	*P* value	Marginal Effect (95% CI)	*P* value
Effect of MNP distribution after 6 months (children 12–18 months of age)	− 0.037 (− 0.087–0.013)	0.147	0.000 (− 0.061–0.062)	0.995
Effect of MNP distribution after 12 months	− 0.049 (− 0.109–0.011)	0.111	0.025 (− 0.025–0.075)	0.715
Effect of MNP distribution after 18 months	− 0.019 (− 0.081–0.042)	0.533	0.015 (− 0.036–0.066)	0.944
R-squared	0.23		0.13	
Observations	6204		6186	

^1^ Intention to treat analysis, and regression estimates from multiple linear models adjusted for gender, age, parenting practice (whether caregiver used toys played with child the previous day, sang to child previous day), BSID tester and county fixed effects. Standard errors are clustered at the village level. MDI data missing for 214 observations (5 in baseline, 101, 88 and 20 in 6 months, 12 months and 18 months follow-up), and PDI data missing for 233 children (7 in baseline, 102, 98 and 26 in 6 months, 12 months and 18 months follow-up)

**Table 10:** Dose–response effects of multiple micronutrient powder provision on Bayley mental and psychomotor development delay

	Mental delay (MDI < 80)^1^		Psychomotor delay (PDI < 80)^1^	
	Marginal Effect (95% CI)	*P* value	Marginal Effect (95% CI)	*P* value
Effect of MNP distribution after 6 months (children 12–18 months of age)	− 0.000 (− 0.001–0.0002)	0.146	− 0.000 (− 0.000–0.000)	0.936
Effect of MNP distribution after 12 months	− 0.001 (− 0.001–0.000)	0.101	0.000 (− 0.00–0.00)	0.323
Effect of MNP distribution after 18 months	− 0.000 (− 0.001–0.000)	0.396	0.00 (− 0.001–0.001)	0.667
R-squared	0.23		0.12	
Observations	6204		6186	

^1^ Intention to treat analysis, and regression estimates from multiple linear models adjusted for gender, age, parenting practice (whether caregiver used toys played with child the previous day, sang to child previous day), BSID tester and county fixed effects. Standard errors are clustered at the village level. MDI data missing for 214 observations (5 in baseline, 101, 88 and 20 in 6 months, 12 months and 18 months follow-up), and PDI data missing for 233 children (7 in baseline, 102, 98 and 26 in 6 months, 12 months and 18 months follow-up)

## Abbreviations

BSID: Bayley scales of infant development
ITT: Intent-to-treat
MNPs: Multiple micronutrient powders
